# Intervention research in health promotion: methodological issues

**DOI:** 10.1093/eurpub/ckac129.074

**Published:** 2022-10-25

**Authors:** A-F Guillemin

**Affiliations:** Research Department, French National Cancer Institute, Boulogne-Billancourt, France

## Abstract

Intervention research in population health focuses on actions carried out by researchers in partnership with those involved in the intervention: health professionals, patients, carers, public policy makers and population communities. The diversity of actors shapes PHIR projects, testifies to the richness of this research and gives it a privileged place to analyse and intervene as accurately as possible in different contexts and populations. It also implies the need for a better understanding of how to intervene by considering the determinants of health in these interventions. PHIR in health promotion in the domain of cancer thus proposes a paradigm shift from describing the problem and its causes to intervention. RISP bases its theoretical anchors on the one hand on the contributions and models of public health, health promotion and human and social sciences and on the other hand, on its own contributions to theorise and build its own theoretical and methodological corpus. This presentation will aim to clarify this paradigm shift and five methodological challenges of PHIR: the imperialism of epidemiology as a research model; the evaluation models of PHIR and their polymorphism; the complexity of the objects of study; the partnership dynamics between researchers and field actors without which research cannot be carried out. Finally, the challenges of publication and valorisation of this type of research. Through concrete examples, participants will be invited to understand the challenges of the RISP methodology, and to analyse different concrete perspectives to address them. The first part will focus on identifying the participants’ representations of the methodological difficulties of PHIR. The second part will be an interactive presentation of 12 minutes, the last 2-3 minutes will be devoted to final questions.

